# Evaluation of a Distal Dorsal Thumb Lesion

**Published:** 2013-04-12

**Authors:** Lily Daniali, Kodi Azari

**Affiliations:** ^a^Department of Surgery, Division of Plastic Surgery, New Jersey Medical School, University of Medicine and Dentistry of New Jersey; ^b^Department of Orthopaedic Surgery/Orthopaedic Hospital and Division of Plastic Surgery, David Geffen School of Medicine at University of California Los Angeles

**Figure:**
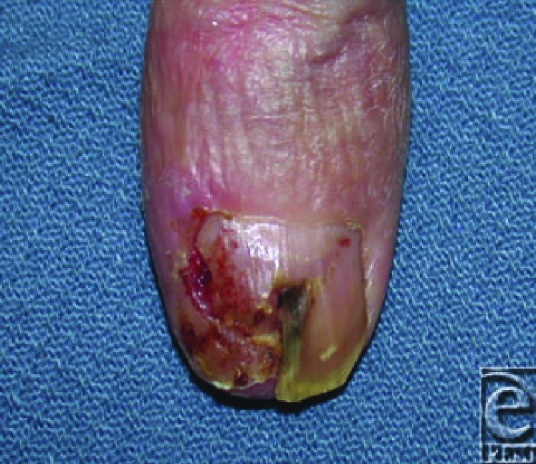


## DESCRIPTION

A 61-year-old right-hand-dominant mechanic was examined for a recurrent thumb lesion present for approximately 5 years. The patient has no history of trauma to the thumb. Over the past 4 years, he was unsuccessfully treated with multiple courses of antibiotics and antifungals, one partial nail plate excision, and one complete nail plate excision for presumed chronic paronychia. The thumb lesion bled intermittently was painful to pressure, scaly, and consumed the lateral eponychial fold. His examination was otherwise unremarkable.

## QUESTIONS

1. **After a thorough history and examination, what is your differential diagnosis?**
2. **What will you do to work up this lesion to determine the correct diagnosis?**
3. **What anatomic feature of the nail bed should raise concern for involvement of underlying structures?**

## DISCUSSION

The appearance of the lesion may mislead the assuming practitioner. While the differential is vast, the etiology of the lesion is most likely either neoplastic or infectious. Possible neoplasms include keratoacanthoma, Bowen's disease (squamous cell carcinoma in situ), invasive squamous cell carcinoma (SCCa), basal cell carcinoma, and metastasis. If the lesion has any associated pigmentation, it is important to rule out melanoma. Acral lentiginous melanoma is the most common melanoma of the nail bed and the most common melanoma among Asians and African Americans. Infectious etiologies include verruca vulgaris, onychomycosis, or chronic paronychia.^1^ To obtain an accurate diagnosis, the plastic surgeon's workup is best guided by the teaching pearl to “culture suspected tumors and biopsy infections.” In addition, the nail bed is intimately associated with the periosteum of the distal phalanx, facilitating occult osseous extension.^2^^,^^5^ Obtaining a radiograph of the hand evaluates for gross bony involvement, but it does not exclude the possibility of microscopic disease.

The lesion was biopsied, and specimens were sent for pathology and fungal culture. The pathological diagnosis returned as “invasive, ulcerated squamous cell carcinoma.” The fungal culture was negative. Radiographs revealed a lack of gross bony involvement. Squamous cell carcinoma is a malignant neoplasm of epithelial squamous cells, and it is the most common tumor of the hand and nail bed. Risk factors include elderly age, male sex, European ancestry, radiation exposure, actinic keratosis, human papillomavirus infection, and chronic scar.^3^

Squamous cell carcinoma in situ may be treated with multiple modalities such as radiation, CO2 laser, chemotherapeutics, and excision. Treatment of invasive SCCa requires surgical excision. Invasive SCCa without periosteal involvement may be treated with Mohs microsurgical serial excision to preserve tissue and digit length for reconstruction. Recent studies have demonstrated cure rates of 95% to 96% with Mohs for the treatment of periungual and subungual SCCa.^4^^,^^5^ Studies have also demonstrated that limited surgical excision results in recurrence rates of up to 56%.^4^ Invasive SCCa with periosteal involvement necessitates a margin of bony excision. Distal phalanx amputation has provided cure rates near 99%.^3^ The efficacy of a limited or partial bony resection of the distal phalanx has not yet been addressed in the literature.
